# Plant Vascular Architecture Determines the Pattern of Herbivore-Induced Systemic Responses in *Arabidopsis thaliana*

**DOI:** 10.1371/journal.pone.0123899

**Published:** 2015-04-16

**Authors:** Abigail P. Ferrieri, Heidi M. Appel, Jack C. Schultz

**Affiliations:** 1 Root-Herbivore Interactions Group, Max Planck Institute for Chemical Ecology, Jena, Germany; 2 Division of Plant Sciences, University of Missouri, Bond Life Sciences Center, Columbia, Missouri, United States of America; Natural Resources Canada, CANADA

## Abstract

The induction of systemic responses in plants is associated with the connectivity between damaged and undamaged leaves, as determined by vascular architecture. Despite the widespread appreciation for studying variation in induced plant defense, few studies have characterized spatial variability of induction in the model species, *Arabidopsis thaliana*. Here we show that plant architecture generates fine scale spatial variation in the systemic induction of invertase and phenolic compounds. We examined whether the arrangement of leaves along the stem (phyllotaxy) produces predictable spatial patterns of cell-wall bound and soluble invertase activities, and downstream phenolic accumulation following feeding by the dietary specialist herbivore, *Pieris rapae* and the generalist, *Spodoptera exigua*. Responses were measured in leaves within and outside of the damaged orthostichy (leaves sharing direct vascular connections), and compared to those from plants where source-sink transport was disrupted by source leaf removal and by an insertional mutation in a sucrose transporter gene (*suc2-1*). Following herbivore damage to a single, middle-aged leaf, induction of cell-wall and soluble invertase was most pronounced in young and old leaves within the damaged orthostichy. The pattern of accumulation of phenolics was also predicted by these vascular connections and was, in part, dependent on the presence of source leaves and intact sucrose transporter function. Induction also occurred in leaves outside of the damaged orthostichy, suggesting that mechanisms may exist to overcome vascular constraints in this system. Our results demonstrate that systemic responses vary widely according to orthostichy, are often herbivore-specific, and partially rely on transport between source and sink leaves. We also provide evidence that patterns of induction are more integrated in *A*. *thaliana* than previously described. This work highlights the importance of plant vascular architecture in determining patterns of systemic induction, which is likely to be ecologically important to insect herbivores and plant pathogens.

## Introduction

Attack by insect herbivores elicits chemical defenses as a critical step in the development of resistance in plants. This induction can occur locally, within a damaged leaf, or in undamaged, system leaves. Many studies have found that systemic defense responses are often highly variable in space and time. Such variation has been attributed to developmental plasticity and plant modularity, to variation in transport of systemic signals, to within-plant genetic variation, and to constraints arising from vascular architecture [1, 2]. Because it can impact herbivore feeding and movement [3] and might slow the evolution of insect adaptation [4] attack-induced variation has even been described as an adaptation [5].

If vascular constraint determines the pattern of plant defense heterogeneity, plant responses to their insect herbivores should often be related to the arrangement of leaves along the stem (phyllotaxy), and the vascular connectivity between these leaves (orthostichy), which are dynamically linked to plant ontogeny [6]. The importance of orthostichy is well known in long-distance nutrient transport, where young, sink leaves are not equally supplied by the same source leaves. Instead, sources preferentially serve sinks that share direct vascular connections along an orthostichy [7]. If resistance-inducing signals travel via the vascular system, we should be able to predict spatial and temporal pattern of defense as it spreads from the site of attack.

Several studies have demonstrated that the spread of induced defense may be related to the degree of connectivity between damaged and undamaged leaves [8–12]. In tomato, the intensity of systemic proteinase inhibitor accumulation correlates well with the degree of vascular linkage between and within leaves, which may be driven by the release of the chemical elicitor, systemin, into the phloem [9, 13–15]. Similar observations have been made in *Nicotiana* spp., where salicylic acid transport [16], the induction of defense-related gene expression [10], and the accumulation of trypsin protease inhibitors [8] all appear related to source-sink relationships. In *Populus* hybrids, local production of phenolics elicited by jasmonates or insect attack depends on movement of photosynthate from sources to directly connected sink leaves [17, 18].

Surprisingly few studies of vascular constraints on systemic responses have employed the model plant *Arabidopsis thaliana*, despite its widespread use in studies of plant responses to insects and pathogens. The relationship between orthostichy and phyllotaxy is well worked out in this plant making it comparatively easy to predict the spatial distribution of systemically induced defenses. *A*. *thaliana* has a 3 + 5 spiral leaf phyllotaxy with a divergence angle between successive leaves of 137.5° [19]. Leaves within an orthostichy are arranged in an approximately vertical line on the stem above each other in the phyllotaxy [20, 21]. Vascular architecture appears to constrain defense responses to pathogen infection to leaves within the orthostichy of an inoculated leaf [20, 21]. We recently used the glucose surrogate, 2-[^18^F]fluoro-2-deoxy-D-glucose, and ^11^C-photosynthate administered to plants as ^11^CO_2,_ to link the movement of potential defense-related substrates to plant architecture in response to wounding and herbivore-related elicitation [22, 23]. Here we showed that radioactivity was most concentrated in the petiole of the radiolabelled, middle-aged leaf and in the younger and older leaves most directly above and below it in the phyllotaxy.

With this information, and our current knowledge of vascular connections within the rosette of *A*. *thaliana*, we used source-sink manipulations and genetic approaches to determine whether plant phyllotaxy produces predictable spatial patterns in two functions associated with systemic defense induction: cell-wall bound and soluble invertases activities, and phenolic accumulation, following herbivore damage to a single leaf. We hypothesized that herbivore-induced changes in invertase activity and phenolic compounds in source and sink leaves are directly attributable to leaf age and location relative to site of damage. If transported carbohydrates serve as building blocks for the production of phenolics in developing sink leaves, we also predicted that intact transport mechanisms would be required as a prerequisite to induction in young leaves.

## Materials and Methods

### Study System

*Arabidopsis thaliana* (Col-0) plants were grown in individual 4cm round pots at 22°C and 62% relative humidity on an 8L:16D photoperiod (180μM PAR) in Metro-Mix 200^TM^ soil (Sun Gro Horticulture, Bellvue, WA) supplemented with 1.8 kg of Osmocote^TM^ slow-release fertilizer (The Scotts Company, Marysville, OH) per cubic meter of soil. Plants were bottom-watered when necessary (approximately every 4 days). Plants were used in the experiments when they were 5 weeks post-germination (contained approximately 15 leaves) and remained in the vegetative state during the experiments.

### Insects

*Pieris rapae* were reared at 24°C on *A*. *thaliana* plants and are the progeny of biological stock originally obtained from Carolina Biological and the Jander laboratory (Cornell University, Ithaca, NY). *Spodoptera exigua* eggs were obtained from Benzon Research (Carlisle, PA) and reared on Beet Armyworm Diet (Southland Products Inc., Lake Village, AR) at 27°C. Post-ecdysial 3^rd^ instar caterpillars were used for all experiments. Insects raised on artificial diet were placed on a pre-feeding diet of *A*. *thaliana* 24h prior to experiment and removed from these plants for a maximum of 3h before the feeding assay.

### Insect Induction Experiments

#### Does plant vascular architecture constrain systemically induced responses elicited by *P*. *rapae and S*. *exigua*

Carbon allocation in *A*. *thaliana* is integrated along a gradient from young leaves at the top, which are entirely sinks, to older leaves at the bottom of the rosette that are weak sinks and/or sources; thus, younger leaves import carbon for growth and metabolism from older sources. Young leaves are particularly responsive to attack, and studies show that they draw carbon from older leaves, stems, and roots [17, 24–26]. That said, we decided to focus on the role of leaves in the middle-aged, transitional leaves located in the center of this gradient, because they act as both sinks and sources and are shared targets of both *P*. *rapae* and *S*. *exigua* feeding on *A*. *thaliana* [27].

**Fig 1:**
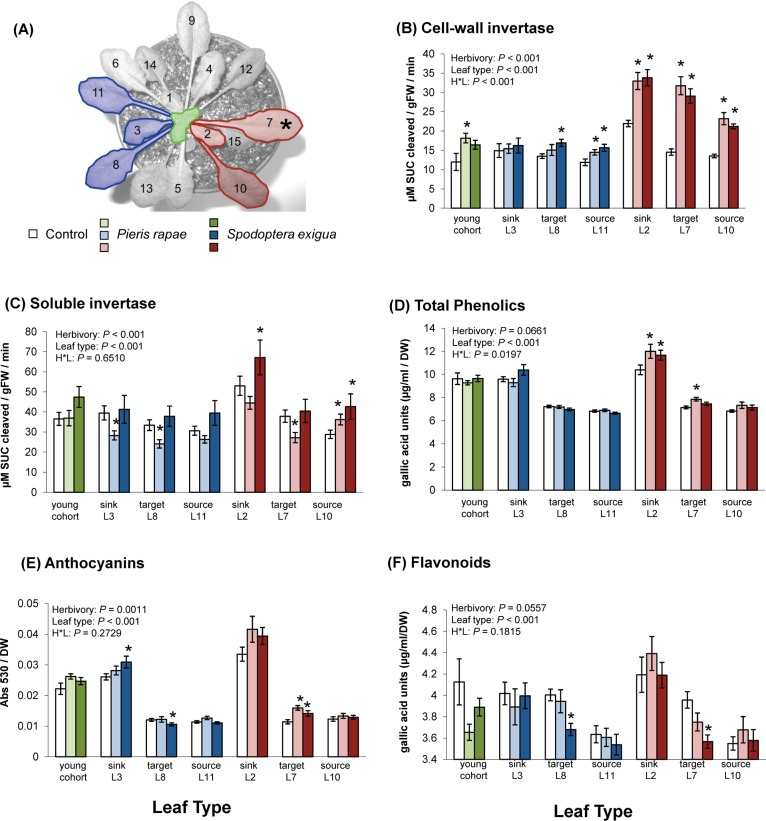
Herbivore-induced changes in foliar invertase activity and phenolic accumulation are determined by orthostichy. (A) *A*. *thaliana* rosette depicting leaves within (red) and outside (blue) of the damaged orthostichy. Asterisk denotes the middle-aged leaf targeted for herbivore damage. Cell-wall (B) and soluble (C) invertase activities were measured 48h following damage *of P*. *rapae* or *S*. *exigua* larva to leaf 7. Foliar accumulation of total phenolics (D), anthocyanins (E), flavonoids (F) were also quantified within and outside of the damaged orthostichy. All bars represent means ±SE. n = 20/treatment group. Results of two-way analysis of variance are shown. Asterisks indicate significant differences between clip-caged control and herbivore elicited plants for a given leaf type (*P* < 0.05 Tukey’s *post hoc* comparisons).

During insect treatments, individual *P*. *rapae* and *S*. *exigua* larvae were confined via clip cage (diameter of 3 cm with nylon mesh screening on top and bottom) to the seventh leaf (L7) from the apex (counting down from the first fully expanded leaf >1mm). Insects were allowed to feed until approximately 30% of the leaf was removed. Clip cages without insects were affixed to L7 of control plants. Toothpicks were used to denote undamaged control leaves after clip cages were removed. Leaves were harvested into liquid N_2_ 48h after insect treatment according to their location within (red, L2, 10) and outside (blue, L3, 8, 11) of the orthostichy of insect-damaged L7 (Fig 1A). Young, undeveloped leaves located at the apex of each rosette (green, Fig 1A) were also pooled and harvested.

#### Is source-to-sink flow of carbon required for defense induction in young, developing leaves

The importance of carbon import for induced phenolic production was examined further in two experiments. The first involved physically removing all source leaves located below young, damaged leaves. The second experiment utilized sucrose transporter mutants.

### Source Leaf Removal Study

**Fig 2:**
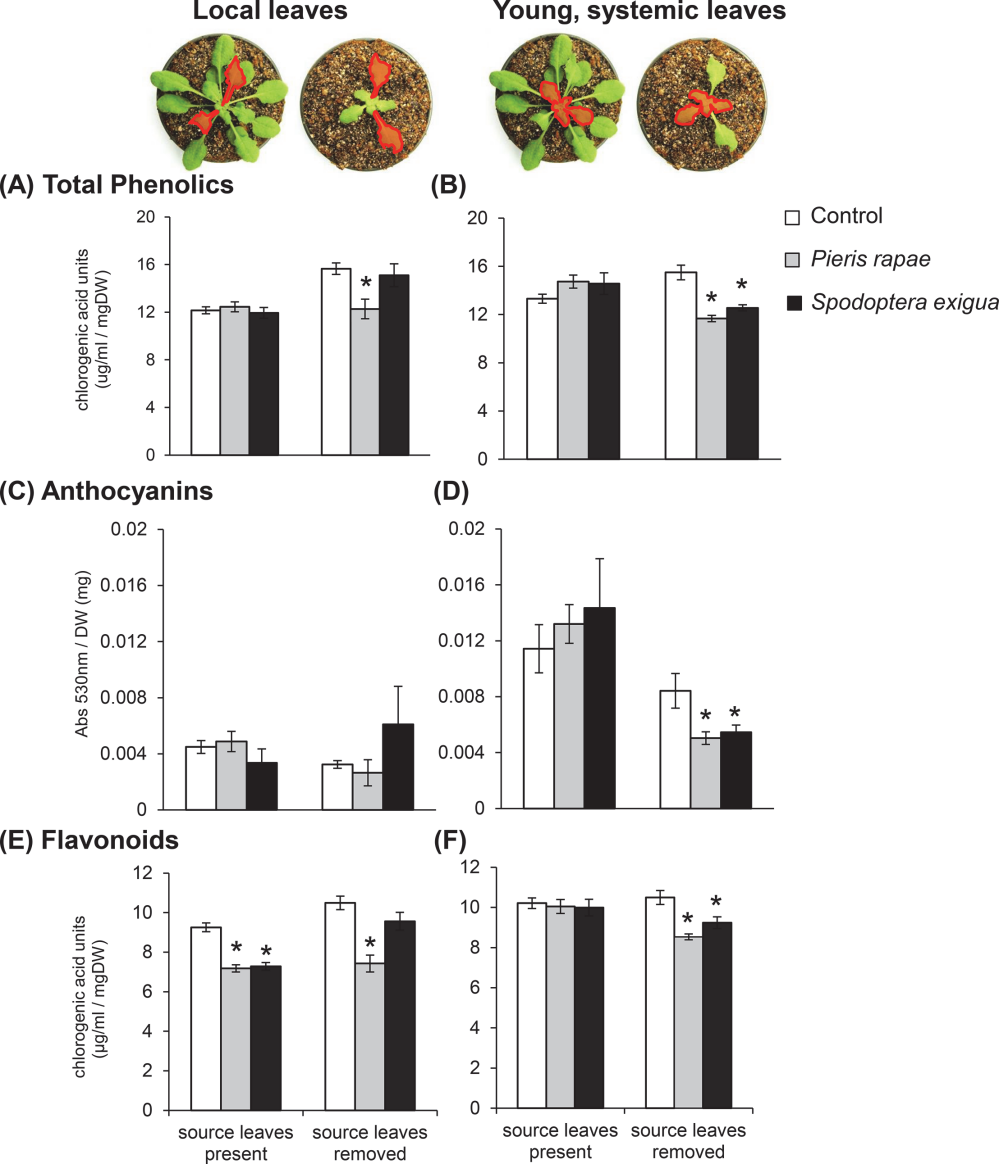
Herbivore-induced phenolic accumulation is dependent on the presence of intact source leaves. The accumulation of total phenolics (A,B), anthocyanins (C,D) and flavonoids (E,F) were measured locally, within herbivore-damaged leaves (A,C,E), and in young, systemic leaves (B,D,F). Bars represent means ±SE. n = 16/treatment group. Results of analysis of variance are summarized in Tables A-C in S1 File. Asterisks indicate significant differences between clip-caged control and herbivore-damaged plants for a given leaf type (*P* < 0.05 Tukey’s *post hoc* comparisons).

The effects of source leaf removal on response to insect treatments were assessed by randomly assigning plants (n = 16) to one of the following treatments: (1) control, with source leaves, (2) *P*. *rapae*, with source leaves, (3) *S*. *exigua*, with source leaves, (4) control, source leaves removed, (5) *P*. *rapae*, source leaves removed (6) *S*. *exigua*, source leaves removed. Source leaves (leaf 4 and greater, see Fig 2) were removed from the appropriate plants at the base of the petiole immediately prior to insect treatments. Larvae were clip-caged to sink leaves 2 and 4 and allowed to feed until they removed approximately 30% of the leaf area. Sink leaves and the young cohort of undeveloped leaves at the top of the rosette were harvested in liquid N_2_ 48h later for phenolic measurements.

### suc2-1 Study

In source leaves of *A*. *thaliana*, photosynthate and presumably other phloem-transported metabolites are loaded apoplastically by the companion cell/sieve-element complex [28]—a process driven by an energy-dependent transport system involving sucrose transporters [29]. Source leaves of the *A*. *thaliana* T-DNA insertion line in AtSUC2 (*suc2-1*, At1G22710), a phloem-specific sucrose transporter gene [29], contain excess starch, and fail to transport radiolabeled sugar efficiently to roots and inflorescences [30]. Work using this genotype not only provides strong evidence in support of apoplastic loading as the primary method for initiating long distance transport in *A*. *thaliana*, but also offers a unique opportunity to test whether phloem loading at the source and subsequent transport to sink tissues are required for induced systemic responses.

AtSUC2-silenced plants were grown side by side with their wild-type (WT) background (Ws-2) for controlled comparisons. Seeds (TAIR) were surface sterilized and germinated on agar plants containing Murashige and Skoog media [31]. Homozygote *suc2-1* seedlings were screened for experiments by their characteristic phenotype following germination on media without sucrose—mutants are visibly smaller than wild-type, with very short primary roots, and translucent cotyledons [30]. For controls, wild-type Ws-2 plants were grown without selection under the same conditions as AtSUC2-silenced plants. Approximately 2 weeks post-germination, homozygous *suc2-1* plants and Ws-2 controls were rescued on fresh media containing 1% supplemental sucrose. Plants that were successfully rescued were transplanted to pots and maintained under the same growth conditions as described above. Homozygous and wild-type plants were approximately 6-weeks post-germination at the time of the experiment. Homozygous plants were generally slower to development than their wild type siblings, and so were slightly smaller at the time of the experiment.

We used a factorial design comprised of 8 plants allotted to one of the following treatments: (1) control, wild-type, (2) *P*. *rapae*, wild-type (3) *S*. *exigua*, wild-type (4) control, *suc2-1* (5) *P*. *rapae*, *suc2-1* (6) *S*. *exigua*, *suc2-1*. As in the source leaf removal experiment, larvae were clip-caged to sink leaves 2 and 4 where they were allowed to feed until 30% of each leaf was eaten. Damaged sink leaves and leaves from the apex of the rosette were harvested in liquid N_2_ 48h later for phenolic measurements.

### Invertase Activity Assays

The activity of cell-wall and soluble invertases was measured in leaves according to previously described methods [22]. Proteins were extracted from fresh frozen and ground tissue in 6 volumes (vol/gFW) of MES buffer (pH 7.0) containing 5mM EDTA, 5% w/v PVPP, serine proteinase inhibitors dithiothreitol (20mM), and benzamidine (2.5mM). Cell-wall bound invertase was assayed in the washed pellets and supernatants were pooled for soluble invertase measurements. Sucrose cleaved by cell-wall and soluble invertase fractions was assayed separately by the generation of glucose monomers during a 15 minute incubation period (pH 4.5, 37°C). Invertase activity was quantified colorimetrically by adding 100 μl of Sumner’s reagent to an equal volume of assay fraction in a 96-well plate (Greiner Bio-One) and incubating at 105°C for 10 min; 33 μl of 40% Rochelle salt was then added and absorbance was recorded immediately at 562 nm. Invertase activity was quantified (μM sucrose cleaved/gFW/min) by standard curve method using glucose as a standard.

### Phenolics Extraction and Quantification

Individual leaves and roots were freeze-dried (4–6mg DW) and ground in a Talboys High Throughput Homogenizer (Troemner, NJ, USA). Phenolics were extracted overnight in 200 μl of 1% (v/v) HCl in methanol at 4°C. An additional extraction with 250 μl distilled water and 500 μl chloroform was used to remove chlorophyll from all samples [32]. Samples were vortexed and centrifuged for 3 min at 3000 × *g*. Relative anthocyanin levels in the aqueous phase were determined spectrophotometrically by measuring absorbance at 530nm. Total flavonoid compounds were also estimated in the same extracts at absorbance 320nm [33, 34]. The concentration of total reactive phenolics present in leaf extracts was determined using the Folin-Denis assay. Standard curves were developed using chlorogenic acid as a standard [35].

### Statistical Analysis

The effect of *P*. *rapae* and *S*. *exigua* feeding on local and systemic induction was assessed with two-way analysis of variance (PROC ANOVA) or general linear models (unequal sample sizes, PROC GLM). These procedures were followed by Tukey’s HSD *post hoc* comparisons to determine individual insect effects relative to controls, as well as differences between the insect species. Normality and equality of variance were verified using Kolmogorov-Smirnov and Levene’s tests, respectively. All statistical analyses were conducted using SAS 9.3 (SAS Institute Inc., Cary, NC, USA).

## Results

### Insect Induction

Measurements of invertase activity and phenolic accumulation demonstrate that herbivore-induced response patterns in *A*. *thaliana* are controlled, in part, by the vascular architecture of the plant (Fig 1). The activity of cell-wall invertase, the form of this enzyme associated with tissue sink strength, was altered by both *P*. *rapae* and *S*. *exigua* feeding, and the magnitude of such changes was dependent on the age and location of a given leaf relative to the site of herbivore damage. Both herbivores increased cell-wall invertase locally (target L7), as well as within orthostichous source and sink leaves (Fig 1B; *P*. *rapae* L2 *P* = 0.0002, L10 *P* < 0.0001; *S*. *exigua* L2 *P* < 0.0001, L10 *P* < 0.0001). *P*. *rapae* feeding also increased cell-wall invertase in the youngest cohort of leaves harvested from the apex of the rosette (*P* = 0.0311), whereas *S*. *exigua* did not (*P* = 0.1714). Cell-wall invertase activity of undamaged source leaves was frequently increased following herbivore damage (Fig 1B; *P*. *rapae* L11 *P* = 0.0400; *S*. *exigua* L8 *P* = 0.0072, L11 *P* = 0.0090). *S*. *exigua* feeding increased soluble invertase within orthostichous, source leaves (Fig 1C; L10 *P* = 0.059) while damage by *P*. *rapae* suppressed soluble invertase locally (L7 *P* = 0.0175), and within undamaged, systemic leaves (L3 *P* = 0.0179; L8 *P* = 0.0148).

*P*. *rapae* feeding increased the accumulation of total phenolic compounds locally (Fig 1D; L7 *P* = 0.0013), as well as in the young leaf directly above the damaged leaf (Fig 1D; L2 *P* = 0.0410). Similar patterns of induction were observed following feeding by *S*. *exigua*; however, only induction in young, orthostichous sinks could be supported statistically (Fig 1D; L7 *P* = 0.1200; L10 *P* = 0.2402; L2 *P* = 0.0441). For both insect species, the accumulation of phenolics in orthostichous sinks co-occurred with visible reddening of leaf petioles during the course of the experiment (Figure A in S1 File). Reddening corresponded with an increase in local anthocyanin production (Fig 1E; L7 *P*. *rapae P* = 0.0260; *S*. *exigua P* = 0.0001). Anthocyanin accumulation was also observed for sink leaves outside of the attacked orthostichy (Fig 1E; *S*. *exigua* L3 *P* = 0.0361) and the youngest cohort of leaves (Fig 1E; *P*. *rapae P* = 0.0600). Feeding by *S*. *exigua* suppressed flavonoid production locally (Fig 1F; L7 *P* = 0.0005) and in middle-aged leaves outside of the damaged orthostichy (Fig 1F; L8 P = 0.0003), while *P*. *rapae* feeding marginally reduced flavonoids within young, undamaged systemic leaves (Fig 1F; *P* = 0.0516).

### Source Constraint on Induction

Removing source leaves alone not only increased total phenolics in *A*. *thaliana* plants, but was found to significantly influence the magnitude and direction of leaf responses following herbivory (Fig 2A and 2B and Table A in S1 File, significant effect of source leaf removal *P* = 0.0373; interaction with herbivory *P* < 0.0001). In undamaged control plants, removing source leaves reduced anthocyanin accumulation in middle-aged (Table B in S1 File, *P* = 0.0020; Fig 2C) and young, systemic leaves (Fig 2D), but did not incur significant changes to flavonoid levels (Fig 2E and 2F and Table C in S1 File, *P* = 0.1436). In plants with intact source leaves, we found that *P*. *rapae* and *S*. *exigua* suppressed flavonoids locally (Fig 2E; *P* < 0.05), but elicited no changes to undamaged, systemic leaves (Fig 2F). We also observed a tendency for young, systemic leaves to accumulate phenolics upon damage by *P*. *rapae* and *S*. *exigua* to intact plants (Fig 2B). In contrast, the concentrations of all phenolic compounds were significantly reduced in young leaves of plants where source leaves were removed following damage by either *P*. *rapae* or *S*. *exigua* (Fig 2B, 2D and 2F).

**Fig 3:**
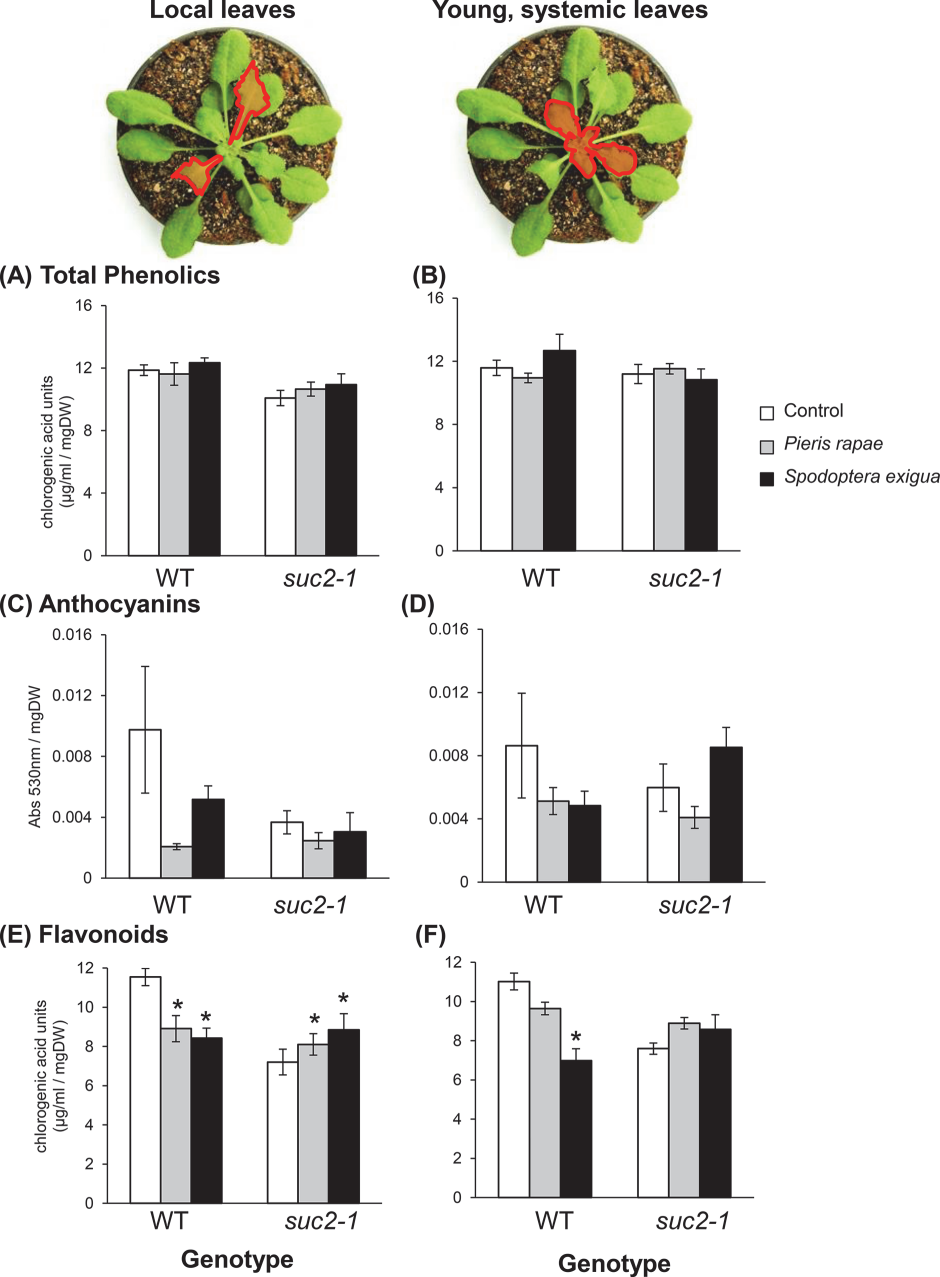
Herbivore-induced changes in foliar phenolic accumulation is independent of intact AtSUC2 function. Herbivore-damaged leaves (A,C,E), and young, systemic leaves (B,D,F) are shown in red. Total phenolics (A,B), anthocyanins (C,D) and flavonoids (E,F) were measured 48h following damage by *P*. *rapae* or *S*. *exigua* larvae to wild-type (WT) and AtSUC2-silenced plants (*suc2-1*). Bars represent means ±SE. n = 8/treatment group. Results of analysis of variance are summarized in Tables D-F in S1 File. Asterisks indicate significant differences between clip-caged control and herbivore-damaged plants for a given leaf type (*P* < 0.05 Tukey’s *post hoc* comparisons).

In general, middle-aged leaves of *suc2-1* plants contained lower levels of total phenolic compounds (Fig 3A and Table D in S1 File, significant effect of genotype *P* = 0.0083), anthocyanins (Fig 3C and Table E in S1 File) and flavonoids (Fig 3E and Table F in S1 File) relative to their WT counterparts. While *P*. *rapae* and *S*. *exigua* did not elicit local or systemic changes to total phenolics (Fig 3A and 3B) or anthocyanins (Fig 3C and 3D), flavonoid levels were significantly suppressed in locally damaged leaves of WT plants (Fig 3E). *S*. *exigua* also suppressed flavonoids in young, undamaged systemic leaves (Fig 3F). In contrast to WT responses, both herbivores elicited an increase in local flavonoid levels within *suc2-1* plants relative to control (Fig 3E).

## Discussion

Here we show that plant architecture generates fine scale spatial variation in the systemic induction of invertase and phenolic compounds. These patterns of induction could be predicted by orthostichous sectors and known vascular connections between source and sink leaves previously identified for *A*. *thaliana* [20–23]. These findings align with those in *Populus* [36], tomato [9], and tobacco [10] demonstrating strong vascular control over patterns of systemic induction. The observed increase in cell-wall invertase in young leaves following herbivore damage is consistent with our previous work demonstrating that the ability of developing leaves to respond defensively to jasmonates, wounding, or herbivory relies on their capacity to induce sink strength for carbon-based resources by increasing cell-wall invertase and importing carbon from orthostichous source leaves [17, 18]. Young sink leaves typically have little in the way of endogenous reserves [37], and often have lower photosynthetic rates compared to mature source leaves, yet are often the most responsive to herbivores [38]. As a result, an increase in invertase activity within sink leaves can act to facilitate greater transport of carbohydrates between source and sink tissues to sustain growth and provide potential building blocks to support the production of carbon-based metabolites, as we have confirmed with labelling studies [17, 18, 22, 23]. Interestingly, we also found that mature source leaves located beneath the damaged leaf also exhibited higher invertase activities following herbivore damage. Given this result, it is also possible that young leaves exhibit induced sink strength by proxy, in which their phenolic accumulation occurs as a byproduct of increased invertase activities in their orthostichous source leaves, creating a need to dispose of excess sugars [39].

The increase in phenolics observed within young, sink leaves in our studies are in agreement with previous findings that an up-regulation of gene transcripts and enzyme activities associated with phenylpropanoid biosynthesis, and the accumulation of phenolic metabolites in leaves following herbivore damage or treatment with jasmonates [17, 40, 41]. Optimal defense theory predicts that the induction of phenolics in young leaves in particular may be favored as these leaves have greater current and future photosynthetic potential for the plant, and are therefore of greater value to potential fitness. In fact, this hypothesis is in agreement with a previous study in *A*. *thaliana* showing that the loss of young leaves incurs more negative effects on reproductive fitness compared to other tissues [42]. In tobacco, young leaves at the apex of the plant are also found to respond most strongly to herbivory with the accumulation of proteinase inhibitor and threonine deaminase transcripts when leaves at a lower nodal position are damaged [10]. This species is also capable of targeting nicotine defense to leaves of higher fitness value, which are not always the wounded tissues [43].

Here we show that within plants, systemic regions orthostichous to sites of herbivore damage accumulate phenolics to a greater extent than non-orthostichous regions. Such constraints in the pattern of induction may be ecologically relevant if they occur at the expense of resistance in non-orthostichous sectors of the plant. Indeed, this has been supported by work in *Solanum dulcamara* where induced resistance in orthostichous leaves coincided with an increase in susceptibility of non-orthostichous leaves to potato beetle, *Lema trilinea* [44]. Since all plants are comprised of modular units defined by plant vasculature, the distribution of signals and resources may also influence where and when herbivores feed. Within-plant and within-leaf resource heterogeneity may also affect the performance and behavior of insect herbivores that exhibit preferences among leaves on a plant. Spatial and temporal variation in leaf quality may force herbivores to make compromises between diet quality and the costs of foraging [3], which may subsequently reinforce defensive traits in plants that are associated with heterogeneity of induced responses [45].

In our study, responses in systemic leaves were not only common, but also showed unique patterns for *P*. *rapae* and *S*. *exigua*. This is consistent with herbivore-specific responses observed in other systems [46, 47] and may be attributed to herbivore-specific salivary components and their ability to elicit different hormonal and metabolic effects in plants [48]. Despite these overall differences, both *P*. *rapae* and *S*. *exigua* appeared to suppress flavonoid accumulation within the leaf they fed on. This is consistent with our previous work observing transcriptional changes in *A*. *thaliana* after herbivory which found that plant responses to both *P*. *rapae* and *S*. *exigua* differed greatly and were frequently weaker or absent in response to the specialist, *P*. *rapae* [49, 50]. Indeed, feeding by *P*. *rapae* and *S*. *exigua* both down-regulated the expression of known transcriptional regulators of flavonoid biosynthesis, including members of the ERF/AP2, MYB, Homeobox, C2H2, BHLH and WRKY gene families within damaged leaves in *A*. *thaliana* [49]. The suppression of secondary metabolites by herbivores is often thought to be mediated by hormone release in plants, including salicylic acid, ethylene and jasmonic acid, which may interact to fine-tune the plant response towards specific attackers [51]. Future studies may explore how such mechanisms may regulate flavonoid levels in this system following herbivore attack.

We found that plants with source leaves removed contained constitutively higher total phenolic levels compared to intact plants. This suggests that the removal of subtending source leaves increased accumulation of carbohydrates into the strongest sinks of the rosette (the young leaves remaining on the plant). As we expected, phenolic induction did not occur in leaves that could not import resources from source tissues below. In fact, in systemic leaves the concentrations of total Folin-reactive phenolics, anthocyanins, and flavonoids were all suppressed following insect damage on plants without intact source leaves. Similar findings have been observed in *Populus* when carbohydrate transport between source and sink leaves was disrupted via steam girdling and sink leaves of girdled plants failed to accumulate phenolics and tannins to the same extent following exposure to jasmonic acid [17, 18].

Systemic induction is likely determined by the impact of phyllotaxy on signaling and material movement through the plant. The type of signal (phloem- or xylem-based, or both) elicited by herbivore feeding determines the spatial and temporal pattern of systemic responses in addition to the impact of vascular architecture itself, and there is evidence for both phloem and xylem transport of signal molecules associated with chemical induction [52]. In *A*. *thaliana*, we found that invertases were commonly induced in source leaves located below the damaged leaf; however this induction did not correspond to changes in phenolic chemistry in any of the classes measured within these mature leaves. This finding sheds light on the directionality of induction and is in agreement with the existence of phloem-based signaling during systemic induction. Yet, the fact that we observed induction of flavonoids in *suc2-1* plants argues against the hypothesis that all responses depend on functional phloem loading. This result is interesting as it suggests that xylem-based signaling or greater mass flow driven by high sieve element and plasmodesmatal densities in developing leaves [53, 54] may facilitate signaling between damaged and un-damaged tissues in *suc2-1* plants.

While vascular architecture was the most consistent predictor of systemic patterns of induction, we found that both a specialist and generalist herbivore frequently induced changes in invertase and phenolic compounds in undamaged leaves located outside of the attacked orthostichy. While plant phyllotaxy links source and sink tissues by a common transport stream, minor connections among other leaves on the stem still exist that may facilitate such responses [28]. Indeed, our recent work demonstrates that the glucose surrogate, 2-[^18^F]fluoro-2-deoxy-D-glucose, is capable of traveling to leaves that are non-orthostichous to the tracer administration site, despite that plant’s highly sectorial vascular architecture, which consists of the leaf petiole that received the radiolabel, and the vertical column of younger and older leaves above and below it [23]. Induction outside of a damaged orthostichy may result from developmentally decreased sensitivity to elicitation (young leaves are more *inducible* than older leaves) or reflect greater connectivity among orthostichies in young leaves whose vascular connections are not fully matured [54]. Given the compact nature of *A*. *thaliana* rosette leaves, it is also likely that within-plant volatile signaling may act as a mechanism to bypass vascular constraints in systemic induction [55]. While this phenomenon has been well-documented in other species, including *Populus* [56] and lima bean [57], further studies are required to determine whether *A*. *thaliana* uses such mechanisms to facilitate signaling across orthostichies.

In summary, we found that systemic responses in *A*. *thaliana* rosettes were best predicted by orthostichy, were often herbivore-specific, and relied, in part, on intact vasculature between source and sink leaves. While induction was most apparent within leaves of the herbivore-damaged orthostichy, our results also provide evidence that induction is sometimes less constrained and more integrated in *A*. *thaliana* than previously described. Nevertheless, future studies which aim to describe herbivore-induced plant responses will benefit from taking into account the potential constraints imposed by plant vascular architecture.

## Supporting Information

S1 File Contains Figure A, Reddening in the petiole of a young leaf 48h following insect damage to the older leaf below it. White arrows point to petioles of young leaves where color differences were evident. Table A, Source leaf removal increases total phenolics in *A*. *thaliana* plants. Summary of 3-way ANOVA comparing total phenolics within two herbivore-damaged leaves and young, systemic leaves of intact *A*. *thaliana* rosettes or those with source leaves removed. Table B, Source leaf removal reduces anthocyanin accumulation in *A*. *thaliana* plants. Summary of 3-way ANOVA comparing anthocyanin accumulation within two herbivore-damaged leaves and young, systemic leaves of intact *A*. *thaliana* rosettes or those with source leaves removed. Table C, Source leaf removal does not influence flavonoid accumulation in *A*. *thaliana* plants. Summary of 3-way ANOVA comparing flavonoid accumulation within two herbivore-damaged leaves and young, systemic leaves of intact *A*. *thaliana* rosettes or those with source leaves removed. Table D, Middle-aged leaves of *suc2-1* plants contain lower levels of total phenolic compounds relative to WT plants. Summary of 3-way ANOVA comparing total phenolics within two herbivore-damaged leaves and young, systemic leaves of wild-type (Ws-2) and mutant (*suc2-1*) lines. Table E, Middle-aged leaves of *suc2-1* plants contain lower levels of anthocyanin relative to WT plants. Summary of 3-way ANOVA comparing anthocyanin accumulation within two herbivore-damaged leaves and young, systemic leaves of wild-type (Ws-2) and mutant (*suc2-1*) lines. Table F, Middle-aged leaves of *suc2-1* plants contain lower levels of flavonoids relative to WT plants. Summary of 3-way ANOVA comparing flavonoid accumulation within two herbivore-damaged leaves and young, systemic leaves of wild-type (Ws-2) and mutant (*suc2-1*) lines.
(PDF)
