# Supplementary material for: Plant Vascular Architecture Determines the Pattern of Herbivore-Induced Systemic Responses in Arabidopsis thaliana
Source: PLoS One. 2015 Apr 16;10(4):e0123899. doi: 10.1371/journal.pone.0123899 (PMC4399992; doi:10.1371/journal.pone.0123899)
Supplement: S1 File — White arrows point to petioles of young leaves where color differences were evident. Table A, Source leaf removal increases total phenolics in A. thaliana plants. Summary of 3-way ANOVA comparing total phenolics within two herbivore-damaged leaves and young, systemic leaves of intact A. thaliana rosettes or those with source leaves removed. Table B, Source leaf removal reduces anthocyanin accumulation in A. thaliana plants. Summary of 3-way ANOVA comparing anthocyanin accumulation within two herbivore-damaged leaves and young, systemic leaves of intact A. thaliana rosettes or those with source leaves removed. Table C, Source leaf removal does not influence flavonoid accumulation in A. thaliana plants. Summary of 3-way ANOVA comparing flavonoid accumulation within two herbivore-damaged leaves and young, systemic leaves of intact A. thaliana rosettes or those with source leaves removed. Table D, Middle-aged leaves of suc2-1 plants contain lower levels of total phenolic compounds relative to WT plants. Summary of 3-way ANOVA comparing total phenolics within two herbivore-damaged leaves and young, systemic leaves of wild-type (Ws-2) and mutant (suc2-1) lines. Table E, Middle-aged leaves of suc2-1 plants contain lower levels of anthocyanin relative to WT plants. Summary of 3-way ANOVA comparing anthocyanin accumulation within two herbivore-damaged leaves and young, systemic leaves of wild-type (Ws-2) and mutant (suc2-1) lines. Table F, Middle-aged leaves of suc2-1 plants contain lower levels of flavonoids relative to WT plants. Summary of 3-way ANOVA comparing flavonoid accumulation within two herbivore-damaged leaves and young, systemic leaves of wild-type (Ws-2) and mutant (suc2-1) lines. (PDF) [file pone.0123899.s001.pdf]

Figure A. **Reddening in the petiole of a young leaf 48h following insect damage to the older leaf below it.** White arrows point to petioles of young leaves where color differences were evident.

Control Insect Damaged

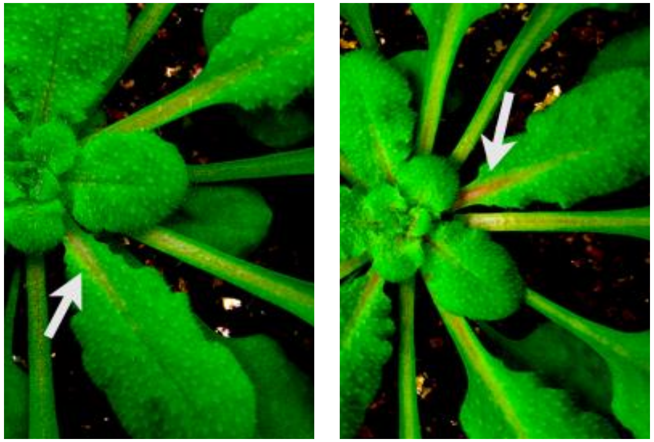

Table A. Source leaf removal increases total phenolics in *A. thaliana* plants. Summary of 3-way ANOVA comparing total phenolics within two herbivore-damaged leaves and young, systemic leaves of intact *A. thaliana* rosettes or those with source leaves removed.

**GLM results**	**Total Phenolics**		
**Source of Variation**	***df***	***F***	**P**
A, Herbivory	2	5.67	0.0042
B, Leaf type	1	2.62	0.1078
C, Source leaf removal	1	4.41	0.0373
A x B	2	0.20	0.8154
A x C	2	14.87	<0.0001
B x C	1	18.71	<0.0001
A x B x C	2	2.47	0.088

Table B. Source leaf removal reduces anthocyanin accumulation in *A. thaliana* plants. Summary of 3-way ANOVA comparing anthocyanin accumulation within two herbivore-damaged leaves and young, systemic leaves of intact *A. thaliana* rosettes or those with source leaves removed.

**GLM results**	**Anthocyanins**		
**Source of Variation**	***df***	***F***	**P**
A, Herbivory	2	0.22	0.7990
B, Leaf type	1	38.11	<0.0001
C, Source leaf removal	1	14.95	0.0002
A x B	2	0.06	0.9437
A x C	2	1.00	0.3715
B x C	1	11.50	0.0009
A x B x C	2	2.10	0.1261

Table C. Source leaf removal does not influence flavonoid accumulation in *A. thaliana* plants. Summary of 3-way ANOVA comparing flavonoid accumulation within two herbivore-damaged leaves and young, systemic leaves of intact *A. thaliana* rosettes or those with source leaves removed.

GLM results	Flavonoids		
**Source of Variation**	***df***	***F***	**P**
A, Herbivory	2	33.79	<0.0001
B, Leaf type	1	45.57	<0.0001
C, Source leaf removal	1	2.16	0.1436
A x B	2	5.76	0.0038
A x C	2	5.87	0.0035
B x C	1	23.54	<0.0001
A x B x C	2	1.91	0.1522

Table D. Middle-aged leaves of *suc2-1* plants contain lower levels of total phenolic compounds relative to WT plants. Summary of 3-way ANOVA comparing total phenolics within two herbivore-damaged leaves and young, systemic leaves of wild-type (Ws-2) and mutant (*suc2-1*) lines.

**GLM results**		**Total Phenolics**	
**Source of Variation**	***df***	***F***	**P**
A, Herbivory	2	0.90	0.4086
B, Genotype	1	7.31	0.0083
C, Leaf type	1	0.34	0.5598
A x B	2	1.37	0.2606
A x C	2	0.08	0.9206
B x C	1	1.34	0.2506
A x B x C	2	0.79	0.4569

Table E. Middle-aged leaves of *suc2-1* plants contain lower levels of anthocyanin relative to WT plants. Summary of 3-way ANOVA comparing anthocyanin accumulation within two herbivore-damaged leaves and young, systemic leaves of wild-type (Ws-2) and mutant (*suc2-1*) lines.

**GLM results**	**Anthocyanins**		
**Source of Variation**	***df***	***F***	**P**
A, Herbivory	2	2.49	0.0891
B, Genotype	1	0.99	0.3221
C, Leaf type	1	1.97	0.1641
A x B	2	1.43	0.2459
A x C	2	0.23	0.7957
B x C	1	0.98	0.3247
A x B x C	2	0.66	0.5203

Table F. Middle-aged leaves of *suc2-1* plants contain lower levels of flavonoids relative to WT plants. Summary of 3-way ANOVA comparing flavonoid accumulation within two herbivore-damaged leaves and young, systemic leaves of wild-type (Ws-2) and mutant (*suc2-1*) lines.

**GLM results**	**Flavonoids**		
**Source of Variation**	***df***	***F***	**P**
A, Herbivory	2	3.70	0.0287
B, Genotype	1	12.59	0.0006
C, Leaf type	1	0.02	0.8770
A x B	2	17.42	<0.0001
A x C	2	1.86	0.1620
B x C	1	1.09	0.2999
A x B x C	2	0.24	0.7846
